# Acute right to left shunt through patent foramen ovale presenting as hypoxemia after myocardial infarction: a case report

**DOI:** 10.4076/1757-1626-2-8878

**Published:** 2009-08-12

**Authors:** Tháràse Franco, Josá Melández, Robert Malkin, Peter Schulman

**Affiliations:** 1 Primary Care Internal Medicine, University of Connecticut263 Farmington Avenue, Farmington, CT, 06030-1234USA; 2 Department of Cardiology, Hospital of Central Connecticut100 Grand Street, New Britain, CT, 06050USA; 3 Department of Cardiology, University of Connecticut263 Farmington Avenue, Farmington, CT, 06032-2202USA

## Abstract

**Introduction:**

This is a report of a 56-year-old man who became hypoxic due to an acute right to left shunt after sustaining a myocardial infarction involving the right ventricle. This case provides the opportunity to review several key pathophysiologic concepts in the setting of acute right ventricular infarction. Although the development of an acute right to left shunt is a rare complication of myocardial infarction, it is important to recognize the diagnosis early in order to prevent life threatening or debilitating clinical sequelae that may result from tissue hypoxia and embolic events. Transesophageal echocardiography is the noninvasive study of choice to confirm the diagnosis. Treatment involves optimization of right ventricular function to minimize shunting. However, medical therapy may provide only temporary relief, and closure of the atrial septal defect should be considered if a clinically significant shunt persists.

**Case presentation:**

A 56-year-old Caucasian man with severe aortic insufficiency presented to the emergency department for evaluation of substernal chest pain. An inferior myocardial infarction was diagnosed by the electrocardiogram and serologic markers. Cardiac catheterization revealed complete occlusion of the right coronary artery as well as a 50-75% stenosis of the left anterior descending artery. Angioplasty of the right coronary artery was performed, but immediate re-occlusion occurred. Subsequently, hypotension and severe hypoxemia developed and persisted despite intubation and mechanical ventilation with 100% oxygen. A significant right-to-left shunt through a patent foramen ovale was diagnosed by contrast transesophageal echocardiogram. Surgical intervention was required and included coronary artery bypass grafting, aortic valve replacement as well as closure of his atrial septal defect.

**Conclusion:**

A right to left atrial shunt is a rare complication of inferior myocardial infarction with right ventricular infarction. The diagnosis should be considered in the presence of inferior myocardial infarction when hypoxemia persists despite administration of 100% oxygen. Early diagnosis and treatment are critical in order to reduce the risk of embolization and to prevent end-organ damage due to hypoxemia.

## Introduction

Patent foramen ovale (PFO) is an anatomic inter-atrial communication with the potential risk for a right-to-left shunt. In utero, the PFO allows for fetal oxygenation; it usually closes within weeks or months after birth. However, in roughly 27% of the general adult population shunting through the PFO can occur [1-5]. The incidence of PFO at autopsy decreases with age, but the size of the defect increases with age [6]. This defect is usually of no clinical consequence. However in the setting of right ventricular failure, high right ventricular diastolic pressure can facilitate right-to-left shunting through a previously silent PFO. The shunting may result in systemic hypoxemia refractory to supplemental oxygen.

Right ventricular MI (RVMI) is a common complication of inferior wall myocardial infarction. The incidence of RVMI with inferior myocardial infarction can be as high as 51% by electrocardiogram criteria [4,7]. The hemodynamic consequences of a RVMI include elevated right ventricular diastolic pressure and depressed cardiac output. Clinical signs include high central venous pressure, clear lung fields and systemic hypotension.

When patients with PFO sustain an IMI associated with right ventricular infarction, the risk of complications is magnified. The potential increase in right atrial pressure can lead to a right-to-left shunt through the patent foramen ovale causing systemic hypoxia. We present a case of acute inferior myocardial infarction complicated by refractory hypoxemia due to the development of an acute right to left inter-atrial shunt through a previously dormant patent foramen ovale.

## Case presentation

**Figure 1.**
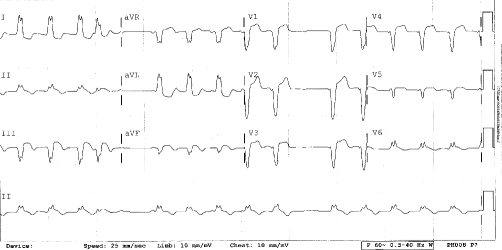
Electrocardiogram showing ST elevations in leads II, II and aVF, diagnostic for acute IMI. Reciprocal changes, ST depression in leads I and aVL, are also observed. In a patient with acute IMI, a right sided electrocardiogram with ≥1mm ST elevation in V4R has a sensitivity of approximately 70% and specificity of approximately 100%. Any patient with an IMI should undergo right sided electrocardiography to evaluate for RVMI as this complication may occur in up to 51% of IMI cases. [4,7] Also of note in this electrocardiogram are conduction disturbances including a (new) left bundle branch block and sinus arrest.

**Figure 2.**
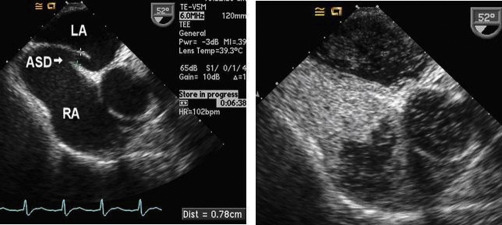
**(a)** Transesophageal echocardiography showing a large atrial septal defect (ASD) between the left atrium (LA) and right atrium (RA). **(b)** Following intravenous injection of agitated saline, contrast is seen crossing the ASD into the left side.

A 56-year-old Caucasian man with a history of severe aortic insufficiency presented to the emergency department with twelve hours of continuous, crushing substernal chest pain. An inferior myocardial infarction was diagnosed by the electrocardiogram (Figure 1) and serologic markers. Aspirin, clopidogrel, a statin and a beta-blocker were administered. At cardiac catheterization, the left ventricular end diastolic pressure (LVEDP) was 20 mmHg, and there was a complete occlusion of the right coronary artery (RCA) as well as a 50-75% stenosis of the left anterior descending artery. Angioplasty of the RCA was performed, but immediate re-occlusion occurred. The subsequent course was complicated by hypotension and severe hypoxemia that persisted despite intubation and mechanical ventilation with 100% oxygen. In an effort to lessen the risk of oxygen toxicity, the lowest FiO2 that maintained the oxygen saturation at least 90% was used. An FiO2 of 0.60 was thus chosen. A PEEP of 10 mmHg was required to maintain oxygenation due to the marked pulmonary congestion caused by severe aortic insufficiency. Transesophageal echocardiography with intravenous microbubbles demonstrated a significant right-to-left shunt through a patent foramen ovale (Figure 2a). The right ventricle was dilated, and the free wall was akinetic. The patient was taken to the operating room for coronary artery bypass grafting, aortic valve replacement and closure of the atrial defect. Immediately following closure of the PFO the patient’s oxygen saturation rapidly improved to 100%. There was a slight decrease in the patient’s cardiac output post-operatively (from 5.4 to 5.0 L/min) following surgery. He was successfully weaned from the ventilator on post-operative day four and the remainder of the hospital course was uneventful.

## Discussion

When patients with MI present with arterial hypoxemia, the causes can include left ventricular failure with pulmonary congestion, preexisting lung disease, pulmonary embolism and intracardiac shunt [6]. In our patient, the LVEDP was elevated during the cardiac catheterization early after the admission. Invasive hemodynamic monitoring at that time revealed an LVEDP of approximately 20 mmHg. The increased LVEDP was likely due to a combination of passive congestion due to the aortic insufficiency and the increased diastolic stiffness due to myocardial infarction.

Later in the hospital course, prior to surgery, the patient’s lungs were clear and the chest X-ray was free of vascular congestion. These findings argue against left ventricular failure with subsequent pulmonary congestion as the cause of his hypoxemia. On the other hand, the patient did develop clinical findings are consistent with right ventricular failure including hypotension with weak peripheral pulses and elevated jugular venous pressure. Right ventricular failure alone would not explain the patient’s hypoxemia.

Pre-existing lung disease and pulmonary embolism were ruled out by high resolution CT scan. While the PaO2/FiO2 ratio (72/0.6 ;= ;120) would meet criteria for ARDS, the diagnosis is safely excluded by the lack of infiltrates on chest X-ray and a PCWP greater than 18 ;mmHg. Hypoxemia that was refractory to intubation raised the index of suspicion for an intracardiac shunt. Also suggestive of intracardiac shunting, the patient developed a systolic murmur in addition to the diastolic murmur of aortic insufficiency. Furthermore, the second heart sound had a fixed split. A right-to-left interatrial shunt was in fact confirmed by contrast transesophageal echocardiogram using agitated saline (micro bubbles).

Echocardiography is the first line imaging procedure for the diagnosis of a PFO and delineation of its morphologic details [6,8,9]. Transesophageal echocardiography produces a higher quality image than transthoracic echocardiography because of the transducer’s proximity to the cardiac structures and because of the ability to use a higher frequency transducer while traversing less tissue [6]. The magnitude of an intracardiac shunt may be quantified by the Qp:Qs ratio, which is the pulmonary to systemic blood flow ratio. This can be measured from echocardiographic data or from cardiac catheterization. In this case, the patient was not hypoxemic at the time of catheterization, and an intracardiac shunt was not a diagnostic consideration. After the patient developed hypoxemia, contrast transesophageal echocardiogram demonstrated the shunt as illustrated in Figure 2b.

Right to left interatrial shunting in acute myocardial infarction carries a substantial risk of morbidity and mortality. Rapid diagnosis and treatment can improve the outcome. Efforts should be directed at optimizing right ventricular function with volume expansion and pressors in order to reduce the degree of shunting. As this condition is rare, there are no randomized controlled trials to guide therapy, but case-based principles support such an approach as the treatment priority [1,2,7].

Revascularization of the culprit coronary artery should be undertaken early to maximize right ventricular function. Pulmonary arterial dilating drugs, such as nitric oxide, and positive inotropic drugs, such as dobutamine, have also been shown to improve right ventricular function. AV pacing has been shown to be beneficial in other cases of PFO shunting with hemodynamic compromise [1]. Positive end-expiratory pressure ventilation should be minimized as it will reduce left ventricular filling [1,2]. There is no evidence to support the use of highly concentrated oxygen; furthermore, its use may expose the patient to oxygen toxicity [2].

Right ventricular function can improve after acute myocardial infarction, and the nonsurgical/non-interventional therapies described above may allow time for a sufficient recovery [4]. However, if a clinically significant shunt persists or if right ventricular function does not recover, then percutaneous or surgical closure of the PFO should be considered [3].

The abrupt closure of a PFO can worsen right ventricular failure due to increased right ventricular volume. In some cases, temporary occlusion of the defect is undertaken percutaneously which allows for an evaluation of how well a permanent closure will be tolerated [2]. When a trial period of temporary closure is not feasible, the decision of whether or not to close a PFO is largely based on clinical judgment that includes assessing the risk of worsening right heart function and of developing a paradoxical embolus. Limited available data suggest that the risk of recurrent neurologic events in patients with a presumed paradoxical embolism is in the range of 4-12%. On the other hand, the rate of procedural and post-procedural complications of percutaneous PFO closure is estimated to be 6% [10]. In this case, the patient was hypotensive and the attempt at revascularization by percutaneous intervention was unsuccessful. Surgical therapy in this patient required coronary artery bypass grafting with aortic valve replacement, and the increased risk of the added procedure (i.e. PFO closure) was small in light of the general clinical picture.

## Conclusion

The clinical syndrome of acute right to left interatrial shunt through a PFO is under-recognized. The diagnosis must be considered when hypoxemia refractory to intubation and mechanical ventilation occurs in the setting of inferior myocardial infarction. Diagnosis must be made quickly, usually with transesophageal echocardiography and contrast echocardiography. Medical treatment is directed at reducing the shunting, but percutaneous or surgical intervention may be required in certain cases.

## Abbreviations

LVEDP: left ventricular end diastolic pressure
PEEP: peek end expiratory pressure
PFO: patent foramen ovale
RCA: right coronary artery
RVMI: right ventricular myocardial infarction
